# Osteogenic Potential of Bovine Bone Graft in Combination with Laser Photobiomodulation: An Ex Vivo Demonstrative Study in Wistar Rats by Cross-Linked Studies Based on Synchrotron Microtomography and Histology

**DOI:** 10.3390/ijms21030778

**Published:** 2020-01-25

**Authors:** Ruxandra Elena Luca, Alessandra Giuliani, Adrian Mănescu, Rodica Heredea, Bogdan Hoinoiu, George Dumitru Constantin, Virgil-Florin Duma, Carmen Darinca Todea

**Affiliations:** 1 Department of Oral Rehabilitation and Dental Emergencies, School of Dental Medicine, “Victor Babeş” University of Medicine and Pharmacy, 300041 Timisoara, Romania; luca.ruxandra@umft.ro (R.E.L.); george.dctin@gmail.com (G.D.C.);; 2 Department of Clinical Science, Polytechnic University of Marche, 60131 Ancona, Italy; a.manescu@univpm.it; 3 Department of Microscopic Morphology/Histology, “Victor Babes” University of Medicine and Pharmacy, 300041 Timisoara, Romania; heredea.rodica@yahoo.com; 4 Department of Pathology, “Louis Turcanu” Children’s Clinical Emergency Hospital, 300041 Timisoara, Romania; 5 Division of Clinical Practical Skills, “Victor Babes” University of Medicine and Pharmacy, 300041 Timisoara, Romania; hoinoiu@umft.ro; 6 3OM Optomechatronics Group, Faculty of Engineering, “Aurel Vlaicu” University of Arad, 310132 Arad, Romania; duma.virgil@osamember.org; 7 Doctoral School, Polytechnic University of Timisoara, 300006 Timisoara, Romania

**Keywords:** Photobiomodulation, bone regeneration, xenograft, collage membrane, synchrotron radiation-based X-ray microtomography, histology

## Abstract

Background: Alveolar bone defects are usually the main concern when planning implant treatments for the appropriate oral rehabilitation of patients. To improve local conditions and achieve implant treatments, there are several methods used for increasing bone volume, among which one of the most successful, versatile, and effective is considered to be guided bone regeneration. The aim of this demonstrative study was to propose an innovative analysis protocol for the evaluation of the effect of photobiomodulation on the bone regeneration process, using rat calvarial defects of 5 mm in diameter, filled with xenograft, covered with collagen membrane, and then exposed to laser radiation. Methods: The animals were sacrificed at different points in time (i.e., after 14, 21, and 30 days). Samples of identical dimensions were harvested in order to compare the results obtained after different periods of healing. The analysis was performed by cross-linking the information obtained using histology and high-resolution synchrotron-based tomography on the same samples. A comparison was made with both the negative control (NC) group (with a bone defect which was left for spontaneous healing), and the positive control (PC) group (in which the bone defects were filled with xenografts and collagen membrane without receiving laser treatment). Results: We demonstrated that using photobiomodulation provides a better healing effect than when receiving only the support of the biomaterial. This effect has been evident for short times treatments, i.e., during the first 14 days after surgery. Conclusion: The proposed analysis protocol was effective in detecting the presence of higher quantities of bone volumes under remodeling after photobiomodulation with respect to the exclusive bone regeneration guided by the xenograft.

## 1. Introduction

Nowadays, dental medicine is confronted with the challenge of solving large edentations complicated with severe bone loss through the usage of implant prosthetic restorations. To make such treatments successful and predictable, specific pre-requisites regarding the bone quantity and quality are required. Alveolar bone defects are usually caused by infections, surgical trauma due to aggressive extraction, periodontal diseases, or accidental trauma [1,2,3]. To improve local conditions and to give the patient the possibility to receive an appropriate implant treatment, several methods and biomaterials for increasing the bone volume have been tested. Bone grafting is the second most frequent tissue transplantation after blood, with over two million procedures reported annually worldwide [4].

One of the most successful bone reconstruction methods is guided bone regeneration. This is considered the most versatile and effective method for alveolar ridge augmentation [5,6,7], offering the possibility of over 5 mm bone gain in the vertical and horizontal aspect [5,8,9,10,11,12]. There are publications that report an increase of even 8.5 mm in height [5,13] by the appropriate usage of this technique. Regarding the bone graft materials, to harvest autogenous bone graft from the patient donor site is still considered the gold standard, even though it has clear limitations regarding the possibility of donor site complications and of subsequent morbidity [1,2,3]. The need to replace autogenous bone with other materials led to the development of allografts, xenografts, and alloplasts [14,15].

Independently of the utilized technique and materials, bone augmentation with a biomaterial and its simultaneous covering with a barrier membrane involve a series of regular biological process of osteo-induction and osteo-conduction. They also involve several cell types and signaling with specific timing [16,17], thus combining the potential of the materials used and their mechanical properties [18]. Hitti and Kerns [19] extensively described the need for a barrier membrane, which enhances new bone formation by preventing the rapid ingrowth of fibroblasts into a bony defect. An important condition for the migration of the osteogenic cells from the bone edges to the defect is sealing the intrabony defect with a membrane. In this case, the rate of osteogenesis exceeds the rate of fibrogenesis [20].

Increased interest is nowadays focused not only on biomaterials but also on the development of methods with stimulatory effects on bone cell proliferation. In this respect, photobiomodulation has shown promising results in stimulating cell proliferation, especially fibroblasts, macrophages and lymphocytes. It also promotes angiogenesis and synthesis of collagen [21,22,23,24]. Specifically, the use of low-level laser therapy (LLLT) for wound healing, nerve injury repair, as well as the reduction of inflammation and pain, was tested shortly after the invention of lasers [25]. Photobiomodulation using laser technology is often referred to as LLLT because the energy delivered to the tissue is much lower than for other laser treatments and it does not produce heating of tissue [25]. In this context, Pinheiro et al. [23] demonstrated that bone irradiated with low-level lasers showed increased osteoblastic and collagen proliferation, as well as new bone formation in comparison with non-irradiated groups. Bone cell and tissue sensitivity to laser irradiation were also reviewed by Barber at al. [26], concluding that laser properties must be carefully chosen to obtain the desired effects. Initially, several in vitro studies were performed with laser radiation on bone cells in culture; different wavelengths were used (690, 830, and 632 nm), with different protocols regarding the duration, the frequency and the dose of radiation [27,28,29,30]. Nevertheless, they all concluded that laser therapy increases the number of more differentiated osteoblastic cells [27,31]. It stimulates cell proliferation, bone nodule formation, followed by osteogenic markers: alkaline phosphatase (ALP) gene expression [28], osteopontin, and bone sialoprotein [23,29]. Another similar study concluded that after 96 h the cell proliferation was obvious, together with higher levels of transforming growth factor-B1, and osteocalcin, whilst ALP levels were not significantly different between different study groups [30]. The same aspects have been investigated throughout many in vivo studies, using different animal models, wavelengths, and irradiation protocols. Most of these studies presented improvements during the early stages of the healing period [32], with significant differences in the calcium hydroxyapatite concentration, as well as in the quantity of newly formed bone and of collagen fibers [33]. Other studies [34] observed increased mineralization in the laser-treated groups and increased levels of calcium, phosphorus, and proteins in comparison to the untreated groups [30].

As the effect of photobiomodulation on empty defects has been well documented in the literature, our interest was focused on the biological interaction between photobiomodulation and the guided bone regeneration technique. In this respect, the Brazilian research group of Pinheiro investigated protocols for improving the bone repair process using photobiomodulation. They used an infrared laser (wavelength 830 nm) on bone defects of rats and rabbits, which were treated following different protocols and using different biomaterials. One of their studies [35] reported an increased amount of newly formed bone after laser usage in rats’ bone defects. Another study revealed that in the early stages of the healing period, differences in bone organization and vascularization were detectable, but after 45 days the differences between the irradiated and the non-irradiated groups became insignificant [36]. Later studies of the same research group [37] investigated the concentration of calcium hydroxyapatite (CHA) around dental implants placed in rabbits’ tibia and concluded that LLLT improved the bone healing, increasing the CHA concentration. However, photobiomodulation in combination with guided bone regeneration needs further study, because of different responses from the hosting tissues.

Thus, the aim of this demonstrative study was to test the effectiveness of an innovative protocol to evaluate the effect of photobiomodulation on bone regeneration process, using rat calvarial defects filled with xenograft, covered with collagen membrane, and then exposed to laser irradiation. The analysis was performed by cross-linking the information obtained by histology and high-resolution synchrotron-based tomography (micro-CT) on the same samples. Comparisons with both the negative control (NC) group (having a bone defect which was left for spontaneous healing), and positive control (PC) group (in which the bone defects were filled with xenografts and collage membrane, without receiving any laser treatment) were made.

## 2. Results

### 2.1. Synchrotron Radiation-Based Micro-Tomography

**Figure 1:**
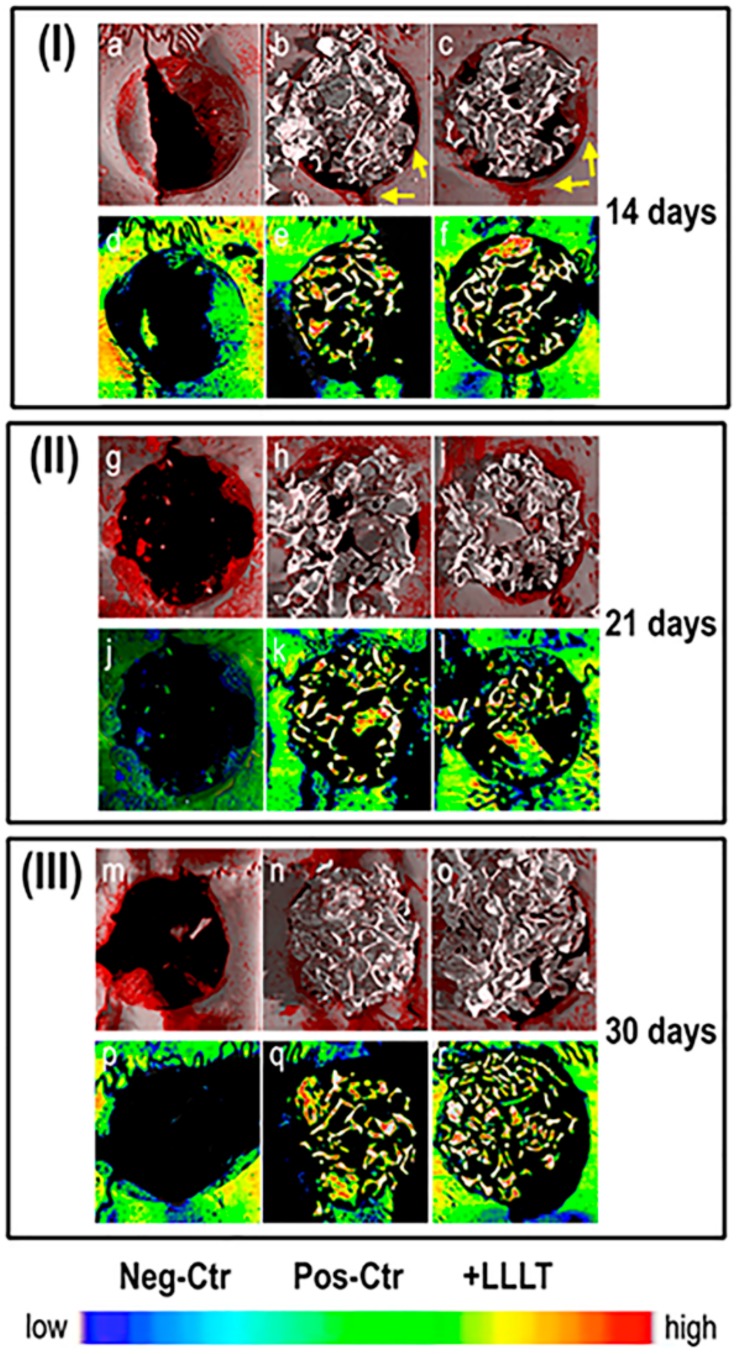
Micro-CT images of repaired sites in representative retrieved samples. ***(*a**–**f**) Group I: samples harvested after 14 days: (**a**–**c**) 3D reconstructions: (**a**) negative control (NC); (**b**) positive control (PC); (**c**) treated with low-level laser therapy (LLLT); (**d**–**f**) transversal sections of the defect: (**d**) NC; (**e**) PC; (**f**) treated with LLLT. (**g**–**l**) Group II: samples harvested after 21 days: (**g**–**i**) 3D reconstructions: (**g**) NC; (**h**) PC; (**i**) treated with LLLT; (**j**–**l**) transversal sections of the defect: (**j**) NC; (**k**) PC; (**l**) treated with LLLT. **(m**–**r)** Group III: samples harvested after 30 days: (**m**–**o**) 3D reconstructions: (**m**) NC; (**n**) PC control; (**o**) treated with LLLT; (**p**–**r**) transversal sections of the defect: (**p**) NC; (**q**) PC; (**r**) treated with LLLT. In 3D reconstructions grey tissue is mature bone; red tissue is bone under remodeling; white tissue is xenograft biomaterial; yellow arrows point to newly formed bone on defect border. In the transversal sections of the defect: white tissue is xenograft biomaterial; colors represent mineralization of the bone proportional to the color map in the bottom. Color map: blue stands for low mass density; red stands for high mass density.

Micro-CT images of repaired sites in retrieved samples are shown in Figure 1. All tissues, except for mineralized bone and residual biomaterials, have been made virtually transparent. Three-dimensional reconstructions of representative samples are shown in Figure 1a–c, Figure 1g–i, and Figure 1m–o, for biopsies harvested 14, 21, and 30 days after surgery, respectively. Moreover, representative transversal sections are respectively reported in Figure 1d–f, Figure 1j–l, and Figure 1p–r, for the same groups of study.

Two aspects are evident in the 3D reconstructions of the represented samples: first, the newly-formed bone (tissue represented in red in Figure 1a–c,g–i,m–o) mainly forms on the borders and not in the center of the defect, and this occurs not only in the NC group, but also in the PC and +LLLT groups (i.e., where the xenograft is present); secondly, with the exception of the +LLLT group, an increase in the volume of newly-formed bone is evident in the period between the 14th and the 21st day after surgery. Moreover, even if the massive presence of the biomaterial prevents a fully reliable evaluation of the observation, it appears that after 14 days from surgery, the thickness of newly-formed bone on the defect borders is higher in the +LLLT group than in the PC group, as indicated with yellow arrows in Figure 1b,c. This event is not as evident for longer amounts of time.

However, from the simple 3D reconstructions and from the representative transversal sections of the repaired defects, volume mismatches in terms of amount of bone under remodeling could not be fully assessed; thus, we proceeded to a volumetric quantitative analysis.

**Figure 2:**
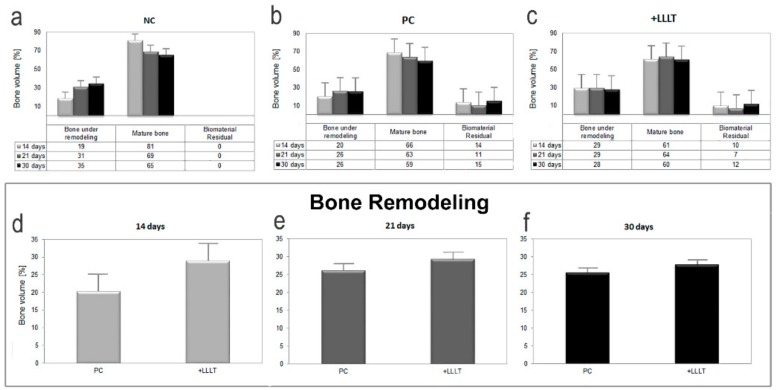
Quantitative morphometric analysis. (**a**–**c**) Mean volume percentages (vol.%) of the different mineralized phases (bone under remodeling, mature bone, and xenograft biomaterial) with respect to the overall mineralized volume: (**a**) NC group; (**b**) PC group; (**c**) +LLLT group. (**d**–**f**) Quantitative volumetric analysis of bone under remodeling portion, after (**d**) 14 days, (**e**) 21 days, and (**f**) 30 days from the surgery. Error bars are indicated.

Indeed, the first step of the study was focused on the investigation of bone microarchitecture and on the evaluation of volume percentages (vol.%) of the different mineralized phases (bone under remodeling, mature bone, and xenograft biomaterial) with respect to the overall mineralized volume. In each harvested sample, a sub-volume fully circumscribing the defect hole was selected, producing the morphometric data reported in Figure 2. As shown in Figure 2a–c, the volume percentages of bone under remodeling with respect to the amount of mature bone increased in time. This trend was observed in all groups except for the laser treated one (+LLLT), where the amount of bone under remodeling was found to be already quite high after 14 days from the surgery.

As observed considering the red phase in the 3D reconstructions of Figure 1a,g,m and based on the quantification reported in Figure 2, samples of the NC group present higher percentages of bone under remodeling than in the other groups, except at the shortest time-point, i.e., 14 days after surgery, when the amount of bone under remodeling was increased by using LLLT by 50% with respect to the NC group and by 45% with respect to the PC group. Moreover, as reported in Figure 2 as well, for more than 14 days from surgery the amount of bone under remodeling was increased by using LLLT by only 10% in comparison to the PC group. One can see, in this respect, an almost unnoticeable increase in the 3D reconstructions of Figure 1.

**Figure 3:**
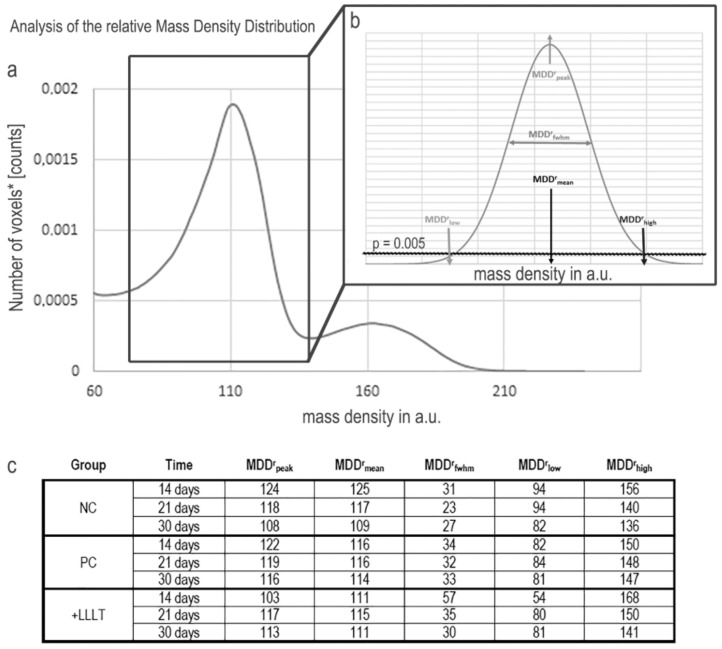
Study of the relative mass density distribution (MDD^r^). **(a)** Portion of the histogram of a sampling biopsy: the peak on the left refers to the overall mineralized bone, the peak on the right refers to the biomaterial (xenograft) used to fill the defect; **(b)** study of the mineralized bone: the parameters investigated with the Roschger approach [38] are indicated. The threshold of *p* = 0.005 has been selected, as a good compromise to maintain a good sensitivity and minimize at the same time potential artifacts due to partial volume effects in the evaluation of MDD^r^_low_; **(c)** parameters that derive from the profile fitting are indicated.

The second step of study was focused on the investigation of the relative bone mineral density distribution (MDD^r^), i.e., on the evaluation of the calcium concentration and distribution (weight%) in the different groups of study. Thus, the same sub-volumes previously investigated for the calculation of volume percentages were also investigated for the MDD^r^ mapping. The concept and results referring to the complete set of indices, derived from the profile fitting, are shown in Figure 3. A sector of the grey-level histogram for a sampling biopsy is shown in Figure 3 Panel a, with the peak on the left referring to the mineralized bone and the peak on the right referring to the xenograft filling the defect. This study was carried out using the Roschger approach [38], which was focused on the mineralized bone portion. The parameters investigated are indicated in Figure 3b. The results, derived from the profile fitting, are listed in Figure 3c. In the NC and PC groups, the peak and the mean values followed a similar trend, which decreases over time. This was not the case for the +LLLT group, where a specific trend was not present. The opposite behavior was detected when considering the FWHM values—a specific trend in time was not present in the NC and PC groups, but in the +LLLT group, where there was a clear decreasing trend over time, with special reference from 14 to 21 days after surgery. Finally, the high value decreased over time in all the groups. Interestingly, after 14 days, the opposite trend was detected considering the FWHM values, while the peak, the mean and the low values were at their maximum in the NC group and minimum in the +LLLT group.

### 2.2. Histology

**Figure 4:**
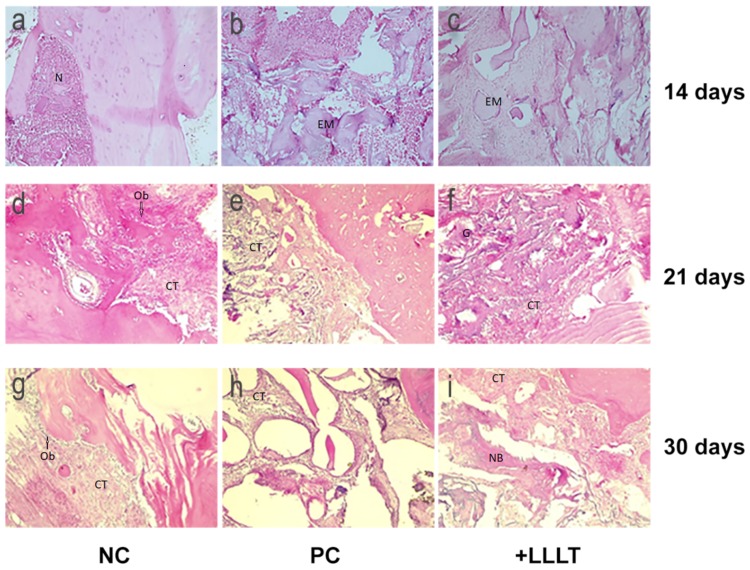
Histologic analysis. **(a–c)** Samples after 14 days of healing: (**a**) group NC, (**b**) group PC, and (**c**) group +LLLT. **(d**–**f)** Samples after 21 days of healing: (**d**) NC group, (**e**) PC group, and (**f**) +LLLT group. **(g**–**i)** Samples after 30 days of healing: (**g**) NC group, (**h**) PC group, and (**i**) +LLLT group. N-necrosis; EM-eosinophilic material; CT-connective tissue; G-granulomas; Ob-osteoblasts. HE staining, original magnification: 10×.

The histological examination, as shown in Figure 4, revealed interesting evidence, with different aspects for groups NC, PC, and +LLLT, depending on the period of healing.

On the examined fragments harvested on Day 14, extensive areas of necrosis and hematic extravasation were identified in the group left for spontaneous healing (NC group, Figure 4a). The other two groups (PC and +LLLT, Figure 4b,c, respectively) showed homogeneous eosinophilic material, a “foreign body” with focal granuloma formation. The +LLLT group fragments revealed well-represented fibrous (young) connective tissue and low inflammatory infiltration compared to the rest of the groups.

Fragments harvested at 21 days after surgery showed a reduction in inflammatory infiltration and foreign body granulomas (Figure 4d–f). These were predominantly located at the periphery, with the fibrous connective tissue embracing eosinophilic material. The NC group revealed fibrotic connective tissue that included optically opaque areas and heavier inflammatory infiltrates (Figure 4d). The +LLLT group showed bone tissue formation, with areas rich in osteoblasts (Figure 4f).

After 30 days of healing, fibrous connective tissue was shown to incorporate homogeneous eosinophilic material and newly formed bone lamellas (Figure 4g–i), the osteoblasts were found in large numbers around the bone lamellae, and the inflammatory process was present, as highlighted by giant multinuclear cells.

## 3. Discussion

Despite the increasing success of the use of photobiomodulation in different areas, there are relatively few reports on their effect on bone repair that are evidence-based. However, due to the positive effects on bone metabolism, the use of photobiomodulation has been encouraged in clinical practice [39]. Renno et al. [40] and Stein et al. [29] showed a significant increase in the proliferation of osteoblasts after laser energy irradiation using an 830 nm diode generating 20 J/cm^2^. In addition, the laser radiation appears to accelerate the process of fracture repair and produce an increase in the volume of the callus formed and an increase in bone mineral density. Effects related to photobiomodulation include increased vascularization, increased osteoblastic activity, organization of collagen fibers, and changes in the mitochondrial and intracellular levels of adenosine triphosphate.

In this context, numerous studies have been also conducted in animal models on the osteogenic properties of different biomaterials, with different outcomes [41,42,43,44,45]. However, photobiomodulation effects, in combination with biomaterials in bone defect repair processes, require further study, as different responses from the host have been found. Most research has been based on animal studies, the most investigated type of animal being the rat, mainly in the tibia bone [46,47,48].

In the present study, as well as in a previous one [49], we evaluated the effect of photobiomodulation on bone regeneration process, using rat calvarial defects filled with xenograft, previously coated with collagen membrane and then exposed to laser irradiation.

In vitro studies such as those conducted by Soleimani [50] and Saygun [51] showed proliferation, stimulation and differentiation of human mesenchymal stem cells into osteoblastic cells in the lased groups. In the first case, a dose of 4 J/cm^2^ (810 nm) was delivered, while in the second study the dose was halved to 2 J/cm2 (685 nm). The effects were also confirmed by Dortbudak [27].

Because bone has limited biological variations to react to stimuli, results may be false or over-interpreted. For example, fibrosis and bone resorption can be the result of biomechanical instability as well as missing osteoconductive properties of a scaffold material. They may also be produced by a rapid material degradation, while biocompatibility is still preserved. Therefore, a step-wise approach is preferred to answer questions of biocompatibility/suitability of a novel biomaterial. Biocompatibility issues should be clarified using a biomechanically unchallenged situation [52], whereas the suitability of a material can be tested in a mechanically challenged defect, modeling long bones after biocompatibility has already been assessed in previous experiments. For both, the cellular reaction (e.g., the presence of inflammatory mono- and poly-nuclear cells) as well as the new bone formation at the surface and within the material are important along with the resorption of the material itself [53]. Reduction of inflammation due to photobiomodulation is one of the most well-accepted effects of light therapy [54], its mechanism being evidenced by a decrease in chemical inflammatory mediators, (prostaglandin E2, leucocytes, tumor necrosis factor TNFα). Photobiomodulation can exert both an anti-inflammatory effect and a pro-inflammatory one, increasing mRNA expression and the protein concentration of anti-inflammatory mediators (IL-10, HSP72), similar to anti-inflammatory steroids [55]. An apparent contradiction has been highlighted between the pro-inflammatory effect of photobiomodulation in in vitro studies and the anti-inflammatory effect found in most of the clinical studies [56].

In our case, the positive effects of photobiomodulation therapy in the initial stages of the bone defect healing were also evidenced by histological examination, which revealed significant differences regarding the presence of inflammatory infiltrate in different study groups: at 14 days, the small amount of inflammatory infiltrate in +LLLT group permits the organization of the young connective tissue, which acts as a precursor of the newly formed bone tissue. When analyzing the 21 days healing period, the reduction of the inflammatory process is more obvious in PC and +LLLT groups. At the same time, as the formation of bone tissue occurs, the +LLLT group shows areas rich in osteoblasts. As the healing period increases, the differences between the analyzed groups in terms of inflammation are reduced, but giant multinuclear cells can still be detected. As a conclusion, it seems that the maximum effects of photobiomodulation appear in the early stages of the bone injury, when a smaller amount of inflammatory infiltrate is associated with increased bone formation.

Our present demonstrative study also showed that photobiomodulation healed the defect better than when only the support of the biomaterial was present. This effect was clearly observed for short-term treatments, i.e., 14 days after the surgery. For longer periods of treatment, the newly formed bone volumes became comparable in grafted defects, with or without laser treatment. Indeed, as shown in Figure 2d–f, the amount of bone under remodeling in defects healed with the xenograft support sensibly increased in volume percentages after the photobiomodulation only in the 14 days group, therefore not for longer periods of time. The presence of higher quantities of bone volume under remodeling in the +LLLT group indicated higher quantities of bone formation, as already seen in previous in vitro studies. Nicolau et al. [57] and Freitas [46] thus showed higher bone cell activity when irradiating rat femur and tibiae defects with 660 and 633 nm lasers. Khadra irradiated calvarial defects with an 830 nm laser and found increased soft and bone tissue in the study group [58]. Weber showed that the healing of autologous bone graft in bone defects was improved through photobiomodulation [59].

Moreover, as demonstrated using the relative mass density distribution (MDD^r^) analysis, after 14 days from surgery the peak, the mean and the low values were at their minimum in the +LLLT group and maximum in the NC group, while the opposite trend was observed for the FWHM values. These data, collectively considered, indicate a wider distribution of mass density after the laser treatment, with a preeminent presence of areas with low mineralization, i.e., with a majority of newly formed bone clusters.

For longer time-points than 14 days (i.e., 21 and 30 days from surgery), when compared with the NC samples (in which the cavities were left empty for spontaneous healing), defects filled with xenografts, i.e., both the PC and the +LLLT samples, presented lower or similar volume percentages of bone under remodeling (i.e., less newly formed bone). This evidence was observed in Figure 1a–c,g–i,m–o, as well as in Figure 2a–c. This was also confirmed by previous studies [41,42]. For example, Takauti [31] studied the bone regeneration process in rat calvariae, with defects filled with three different types of biomaterials: two xenografts (deproteinized bovine bone) and one allograft (biphasic calcium phosphate). After eight weeks of healing, they found a greater amount of newly formed bone when using alloplastic materials, while cavities filled with deproteinized bovine bone presented higher amounts of residual graft, probably due to its slow resorption. When compared with control cavities, which were left empty for spontaneous healing, they concluded that defects filled only with blood clot presented more newly formed bone than cavities filled with xenograft, and a similar amount to the defects filled with alloplastic material. Their results were also confirmed by Rokn [42].

The evidence that defects left for spontaneous healing presented already after three weeks of healing (21 days group) more newly-formed bone than cavities filled with xenograft could most likely be caused by a certain delay of the healing process in the presence of biomaterials. We observed this delay in several of our previous studies; specifically, we observed that regenerative kinetics in in vitro cultures on different biomaterials showed that the bioresorption of the scaffold is more accentuated up to the second week of culture, while bone regeneration is delayed in time, most likely because cells growing onto the scaffold took longer time to adhere and then to begin proliferating [60,61,62].

**Figure 5:**
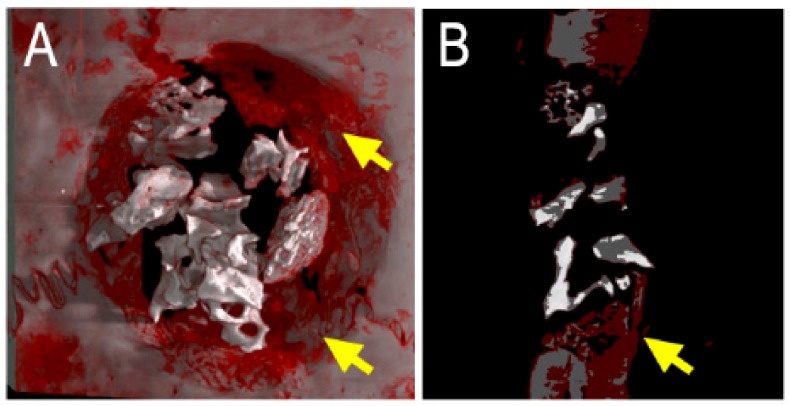
Micro-CT images of repaired sites in representative retrieved sample, evaluated at 30 days postoperatively. The borders of the defect have not been covered with bovine bone graft, thus being directly exposed to laser radiation. A great amount of newly-formed bone can be observed at the periphery of the defect, as indicated with yellow arrows. (**A**): 3D reconstruction; (**B**): transversal section. Grey tissue is mature bone; red tissue is bone under remodeling; white tissue is xenograft biomaterial.

Moreover, in the present study, the biomaterial may have exerted a shielding action in respect to photobiomodulation effects on cells, inhibiting the same regenerative action of the laser treatment, as shown in Figure 5. In the study carried out by Rokn [42], the 3D analysis based on cone-beam tomography showed no filling in the center of the spontaneously healed cavities, revealing that newly formed bone was concentrated only at the edges of the defect. Therefore, it becomes important to favor osteo-conduction and the migration of cells from the defect border, using as filler a biomaterial that adheres to the defect walls without carrying out any barrier and shielding action to cell migration or to the possible regenerating action of laser treatments.

However, in our study, the quantitative volumetric analysis of bone under remodeling at the three time-points (14, 21 and 30 days from surgery) showed better healing when photobiomodulation was applied on the grafted defect than in cases where the grafted defect did not receive laser treatment. This effect was particularly evident for the shortest considered period of time, i.e., 14 days after surgery. Thus, these observations obtained using our innovative protocol of analysis highlight the positive effects of laser therapy on bone regeneration process, which increase the quantity of newly formed bone. It also suggests possible interactions with the grafting materials that could influence our future experimental follow-ups.

In the present study, we selected 14, 21, and 30 days from surgery as time-points for the analysis. The rationale behind this choice was motivated by the fact that, when establishing follow-up periods of bone regeneration in rat calvarial defects, the metabolic rate of the Wistar rat must be considered: the smaller the animal, the higher the metabolic rate compared to that of a human: 30 days of a man’s life correspond to one day of rat’s life [63]. This means that shorter observation periods to obtain data sampling are usually required when small animals are used instead of larger ones, because they heal faster. Long periods of observation would potentially demonstrate that both test and control groups reach an advanced/complete healing of the defect, failing to disclose the beneficial potential of a biomaterial [64,65].

Therefore, considering the short-term laser treatment benefits shown in the present study, we plan to proceed in future experiments by stopping the +LLLT after 14 days and allowing the regeneration to occur beyond this period in the absence of +LLLT, checking the results with our experimental protocol. Moreover, another experimental follow-up will be based on additional +LLLT doses at periods of time shorter than 14 days, examining bone regeneration beyond this period.

## 4. Materials and Methods

### 4.1. Animal Model and Groups of Study

The experimental protocol was approved by the Ethics Committee of the “Victor Babes” University of Medicine and Pharmacy of Timisoara (No. 129 of the 8th December 2016). The study, in order to minimize the number of sacrifices, included 24 Wistar rats with an average weight of 287 g (range 247–312 g), which were randomly divided into 3 study groups: (1) the negative control (NC) group, having a bone defect which was left for spontaneous healing; (2) the positive control (PC) group, in which the bone defects were filled with xenografts and collage membrane, without receiving any laser treatment; and (3) the test (+LLLT) group, with bone defects filled with xenografts, with collagen membrane and receiving low level laser irradiation every 48 h. The Animal Facility of the “Victor Babes” University of Medicine and Pharmacy of Timisoara provided and housed all the animals in a temperature-controlled environment. The rats received water and standard laboratory animal chow ad libitum. A 12 h light–dark cycle was maintained throughout the experimental protocol.

### 4.2. Surgical Procedure

In the first session, the animals were anesthetized (5% Isofluran and O_2_ at 1 L/min, for induction in the anesthesia chamber, and after that, a facemask of 1% Isofluran and O_2_ at 1 L/min was delivered), the region around the scalp was shaved and antisepticised with betadine in order to perform the surgical procedure. This consisted of creating a calvarial circular defect of 5 mm in diameter, using a trephine bur and continuous irrigation with saline solution. To obtain the precise position of the defect related to skin landmarks, a surgical plastic guide was used. After the defect was created, each animal was treated corresponding to the study group to which it was assigned. The animals from the NC group were sutured and received no other treatment, to be able to observe the spontaneous healing of the bone defect. The animals from the PC and +LLLT groups received bovine bone graft (NuOss^®^ natural cancellous and cortical bone matrix, ACE Surgical Supply, USA) into the defect and collagen membrane (ACE RCM6^®^ Resorbable Collagen Membrane, ACE Surgical Supply, USA) covering the defect, thus ensuring the proper conditions for guided bone regeneration to take place. All animals were sutured in a two layers manner and were kept postoperatively in the same conditions: 22 ± 5 °C temperature and at a 50% ± 5 humidity, following an antibiotic prophylaxis treatment (Cefazolin 15mg/kg + Gentamicin 1.5 mg/kg) and daily clinical examination with evaluation of the general clinical status (heart rate, respiratory rate, body temperature, wound appearance and healing of the incision, posture and locomotion).

### 4.3. Photobiomodulation Protocol

Photobiomodulation using laser irradiation was performed every 48 h to animals from the +LLLT group, with a gallium-aluminum–arsenide laser (GaAlAs) (IRRADIA Mid-Laser^®^, Stockholm, Sweden, center wavelength of 808 nm, optical power of 450 mW). Laser irradiation was applied in four peripheral opposite points and in one central point of the defect, with the help of the surgical guide, with a frequency of 3800 Hz, 450 mW, 17 s per point, 18.9 J per treatment session. The photobiomodulation parameters are provided in Table 1.

### 4.4. Samples Collection

The results of the study were assessed by harvesting bone samples from the animals at three different time points: after 14 days, 21 days, and 30 days from the surgery, in order to evaluate the regeneration process versus time. The specimens were all standardized at the 1 × 0.6 × 0.2 cm^3^ dimensions and were fixed in no less than 9 times their own volume of 10% formalin. After harvesting the samples, the rats were euthanized using a Thiopental overdose. All samples were kept in formalin and then investigated with high-resolution X-ray tomographic (micro-CT) and histological examinations.

In order to obtain objective results, the samples were randomly numbered with the aim to be identified only by the scientists involved in the analyses of the results. Although it was obvious which were the negative control samples (empty defects), the healing period was unknown. For the rest of the samples, the aspect was similar, all containing grafting material and the numbering did not permit the assessors to know neither if the sample received photobiomodulation treatment, nor the healing period.

### 4.5. Synchrotron Radiation-Based Micro-Tomography

X-ray microtomography (micro-CT) of the samples was performed at the SYRMEP beamline of the ELETTRA synchrotron facility (Basovizza (TS), Italy). The samples were investigated using the following settings: isometric voxels with an edge size of 9 µm; exposure time of 1 s/projection; and X-ray beam energy of 19 keV. The sample–detector distance was set to 300 mm, enabling us to measure the phase-contrast signal. The phase-contrast configuration differs from conventional tomographic imaging that is based solely on attenuation contrast. Indeed, the refraction of the X-ray beam passing through each tissue is described by the refractive index, *n(r) = 1 − δ(r) + iβ(r),* where *δ* is the refractive index decrement and *β* is the attenuation index. As *δ* is sensibly larger than *β*, the phase-contrast approach is much more sensitive than the absorption approach. The refractive index decrement *δ* is proportional to the mean electron density, which in turn is nearly proportional to the mass density *ρ* (expressed as mg/cm^3^).

In some cases, specifically in weakly absorbing samples or in samples consisting predominantly of a single phase, the real and imaginary parts of the refractive index are proportional to each other, i.e., δ(r) = ε·β(r), where ε does not depend on the spatial coordinates [61,62]. As our samples consisted mainly of mineralized bone, i.e., a single phase with a spatially varying density, and considering the sample-detector distance in the near field regime [66], the previous approximation is valid, and the *δ/β* ratio has been set to 200. The complete tomographic reconstruction was performed using the SYRMEP Tomo Project (STP) open source software [67].

Afterwards, the VG Studio MAX 1.2 software (Volume Graphics, Heidelberg, Germany) was used to generate 3D images, where grey levels were proportional to the mass density *ρ*. The Scatter HQ algorithm with an oversampling factor of 5.0 was used to image the 2D sections and the 3D reconstructions. Different peaks in the gray level scale represent different phases within the samples; the volume of each phase was obtained by multiplying the volume of a voxel (~730 µm^3^) by the number of voxels underlying the peak associated with the relevant phase. A manually set threshold was applied to the histograms to separate the bone under remodeling from the mature bone, and the mature bone from the scaffold phase. The thresholds were set to 108 and 147, respectively.

A morphometric analysis was performed to evaluate the volume percentages (vol.%) of the following phases with respect to the overall mineralized volume: bone under remodeling, mature bone, and scaffold.

Moreover, the refractive index *n* signal, linearly proportional to the mass density, was exploited to compute the relative bone mass density distribution (MDD^r^) of each sample. As we recently proceeded in other studies [68], the MDD^r^ parameters were calculated with strict reference to the mineralized bone portion of the histograms, with the intensities normalized, for each sample, by the area under the curve. The absolute values of bone mass density (calcium concentrations – weight%) could not be retrieved: in fact, *n* might be biased due to the constant ratio *δ/β* used in the Paganin phase retrieval [69]. However, as the samples were comparable in terms of size and composition, the relative differences in mass density distribution between them could be evaluated. Thus, the superscript *r* was used to indicate relative values for all bone mass density distribution parameters. Based on the Roschger approach [38], the following parameters were extracted: MDD^r^_mean_ (mean relative mass density), MDD^r^_peak_ (most frequent relative mass density), MDDr_low_ (0.5th percentile) and MDD^r^_high_ (99.5th percentile), and MDD^r^_fwhm_ (full width at half maxima of the distribution). We arbitrarily selected the threshold of *P = 0.005* as a good compromise between maintaining a good sensitivity for low and high values in the MDD^r^ and reducing possible artifacts originating from the partial volume effect (when evaluating the MDD^r^_low_). This post-processing calculation of the MDD^r^ parameters was done using the PeakFit software (Systat Software, San Jose, CA, USA).

### 4.6. Histology

The tissues obtained were fixed in 10% formalin solution, followed by a moderate descaling agent. The paraffin blocks resulting from the processing were used to make additional sections (thickness of 4 µm, Thermo Scientific™ HM 355S Automatic Microtome, Waltham, MA 02451 USA ) that were stained with hematoxylin and eosin (HE) and were further on examined under a Leica DM750 microscope (Leica Microsystems, Wetzlar, Germany).

## 5. Conclusions

We demonstrated that photobiomodulation therapy is effective in short periods; laser doses administrated to the defects beyond 2 weeks after surgery appeared to be not very effective. A possible shielding action of the xenograft on the laser action on cells was hypothesized and will be verified through future studies. This effect may be combined with a bone regeneration delay in the presence of biomaterials, as already documented in previous studies. Thus, in support of this hypothesis, several authors suggested the same conclusions [33,70], finding that the positive effects of photobiomodulation on bone healing are more obvious when applied intraoperatively, directly to the bone defect, prior to the grafting procedure [33].

In our demonstrative study, micro-CT allowed us to achieve new and relevant information, although a limited number of rats was included in the study. This sample size would have most likely not been sufficient in other experimental protocols, exclusively based on histology. The power of our protocol lies in the 3D nature of micro-CT analysis, based on the stacking of 1000 successive 2D sections (each with a thickness of about 9 μm), mapping the entire sample. This is of paramount importance, allowing us to minimize the number of rat sacrifices, in full respect to ethical international rules. Our previous studies, using the same method of evaluation, also showed the capacity of micro-CT technique to play a fundamental role in the advanced characterization of laser-treated sites [71]. Another technique which can be successful for such a research strategy is optical coherence tomography (OCT) [49,72,73,74]: its advantage is that it can be applied for in vivo assessments, using handheld scanning probes in the oral cavity [75,76]. Moreover, good agreement between OCT and micro-CT analyses was found in our previous studies [77].

In general, the interaction between laser radiation and different types of tissues remains a major concern when establishing clinical protocols. Although numerous studies have been conducted on the effects of photobiomodulation, their comparison is difficult because of the different biomaterials, the variations in laser energy, dose, and duration.

## Figures and Tables

**Table 1:** Photobiomodulation parameters in the experimental protocol.

Manufacturer	IRRADIA Mid-Laser^®^ Stockholm, Sweden
Model Identifier	MID-laser; Serial no 8110131-4
Year Produced	2007
Number and type of emitters	Gallium-Aluminum–Arsenide laser (GaAlAs) laser
Wavelength and bandwidth	808 nm
Pulse mode	CW
Beam spot size at target	1 cm^2^
Irradiance at target	450 mW/cm^2^
If pulsed peak irradiance	450 mW/cm^2^
Exposure duration	17 s per point, 85 s per session
Radiant exposure	24.075 J/cm^2^
Radiant energy	18.9 J
Number of points irradiated	5
Area irradiated	1 cm^2^
Application technique	Photobiomodulation was applied to the skin covering the surgical defect in four peripheral opposite points and in one central point of the defect (the size of the defect was 5 mm in diameter), using a plastic surgical guide
Number and frequency of treatment sessions	Surgery day and every 48 h after the surgery, for 14 days, 21 days, and 30 days respectively
Total radiant energy over entire treatment course	151.2 J for the 14 days group; 226.8 J for the 21 days group; 302.4 J for the 30 days group

## Abbreviations

LLLT	Low level laser therapy
3D	Three-dimensional
MDD^r^	Relative bone mineral density distribution
Micro-CT	Microtomography
